# The Use of Soy Isoflavones in the Treatment of Prostate Cancer: A Focus on the Cellular Effects

**DOI:** 10.3390/nu15234856

**Published:** 2023-11-21

**Authors:** Hans Van der Eecken, Steven Joniau, Charlien Berghen, Kato Rans, Gert De Meerleer

**Affiliations:** 1 Department of Urology, University Hospital Brussels, 1090 Brussels, Belgium; 2 Department of Urology, University Hospitals Leuven, 3000 Leuven, Belgium; steven.joniau@uzleuven.be; 3 Department of Radiation Oncology, University Hospitals Leuven, 3000 Leuven, Belgium; charlien.berghen@uzleuven.be (C.B.); kato.rans@uzleuven.be (K.R.); gert.demeerleer@uzleuven.be (G.D.M.)

**Keywords:** isoflavone, genistein, daidzein, equol, prostate cancer

## Abstract

A possible link between diet and cancer has long been considered, with growing interest in phytochemicals. Soy isoflavones have been associated with a reduced risk of prostate cancer in Asian populations. Of the soy isoflavones, genistein and daidzein, in particular, have been studied, but recently, equol as a derivative has gained interest because it is more biologically potent. Different mechanisms of action have already been studied for the different isoflavones in multiple conditions, such as breast, gastrointestinal, and urogenital cancers. Many of these mechanisms of action could also be demonstrated in the prostate, both in vitro and in vivo. This review focuses on the known mechanisms of action at the cellular level and compares them between genistein, daidzein, and equol. These include androgen- and estrogen-mediated pathways, regulation of the cell cycle and cell proliferation, apoptosis, angiogenesis, and metastasis. In addition, antioxidant and anti-inflammatory effects and epigenetics are addressed.

## 1. Introduction

Globally, a geographical difference is observed in prostate cancer (PCa) incidence and mortality. East Asia has a lower PCa incidence than Western countries. These differences are associated with a migration effect: migrant Japanese men are at the same risk of PCa as Native Americans living in the United States [1]. These differences have been repeatedly associated with dietary habits. Epidemiological studies suggest an inverse relationship between soy isoflavone intake and PCa risk [2,3]. A large number of preclinical studies have proposed some elucidating mechanisms in the carcinogenesis of PCa by soy isoflavones. However, clinical data to confirm this preclinical evidence are currently lacking. Extensive studies and reviews have already been published on the mechanisms of action of soy isoflavones [4,5,6,7,8].

The disease characteristics of PCa, with high incidence, long latency, the availability of tumor markers (prostate-specific antigen (PSA)), heterogeneous risk groups, and the presence of pre-neoplastic lesions, make PCa suitable for chemoprevention by soy and other nutraceuticals [9].

This review focuses exclusively on the cellular effects and the potential clinical use of soy isoflavones, in particular, genistein, daidzein, and its metabolite equol.

## 2. Soy—One Word, Different Worlds

**Figure 1:**
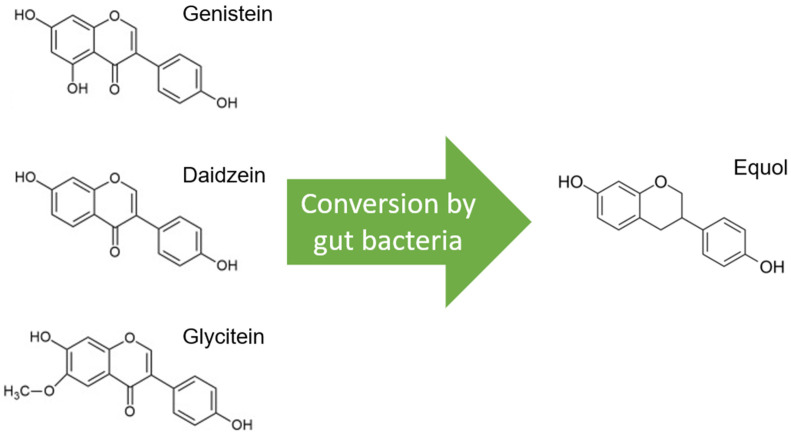
Molecular structures of the isoflavones genistein, daidzein, and glycitein, which are very similar, but note the distinct O-methyl group in glycitein. Only daidzein is converted to equol by gut bacteria.

Soybeans are the richest source of isoflavones, and when soybeans are fermented, isoflavone aglucon is produced through the removal of a glucoside group [10]. The most well-known aglucones are genistein, daidzein, and glycitein. Glycitein differs slightly from genistein and daidzein due to a separate O-methyl group and represents only 5–10% of total isoflavones (Figure 1).

Glycitein has, therefore, been less studied and is therefore excluded from this review, although its beneficial effects have already been described, especially in gastrointestinal cancers and breast cancer [11]. Only daidzein, and not genistein or glycitein, is converted to equol by the microflora in the gut, and this is estimated in only 20–35% of the Western population versus 60% of the Asian population (Figure 1) [12]. Only S-equol, an enantiomer with selective affinity for estrogen receptor β (ER-β), is made by these gut bacteria, in contrast to R-equol, which has more affinity for estrogen receptor α (ER-α) [13,14]. Equol differs from genistein and daidzein in chemical characteristics, and consequently, equol has some other features, such as greater antioxidant activity [15].

The biological effects of genistein, daidzein, and equol are expressed via interaction with multiple and complex cellular pathways. There is interaction with androgen- and estrogen-driven pathways, cell proliferation and cell cycle, angiogenesis, and metastasis. In addition, these molecules have anti-inflammatory and antioxidant properties and possess potential anticancer epigenetic activity. The “characteristics of cancer” were used as a conceptual guide in this review [16]. A concise summary of the molecular mechanisms of action of genistein, daidzein, and equol in prostate cancer is shown in Table 1.

It is important to stress that most of the results in this review were obtained with in vitro studies, where it is generally believed that plant molecules such as isoflavones may have a more pronounced effect when applied directly to cell culture versus in vivo. Therefore, there is also a difference in dosage, and smaller doses are used on average in vitro, ranging from low concentrations (0.1–5 µM) to medium (10–50 µM) and higher (200 µM) concentrations. There may also be a difference depending on the cell lines used. In contrast, animal models use different dose levels, ranging from low (5 mg/kgBW to 20 mg/kgBW) to higher doses (100 mg/kgBW to 250 mg/kgBW) or even more, spread over one or more intakes per day (BW = body weight). The above comment applies to the different sections highlighted below, and therefore, it is always noted whether the results were reported in cell lines or in vivo.

## 3. The Modification of Androgen- and/or Estrogen-Mediated Carcinogenesis

**Figure 2:**
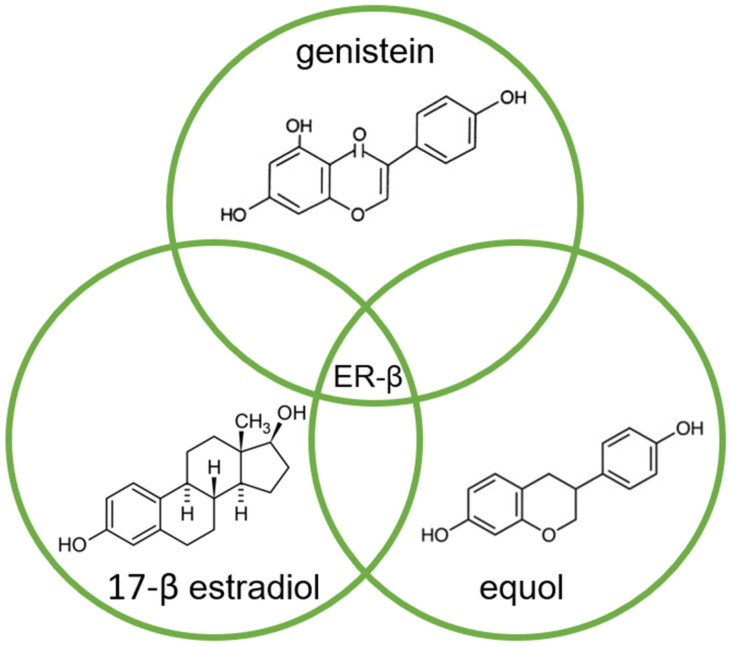
Molecular structures of genistein, equol, and 17-β estradiol, which share a common feature of high affinity for binding to the estrogen receptor (ER-β), due to their high similarity.

Of the two known estrogen receptors in humans (ER-α and ER-β), ER-β is predominantly found in the prostate [83]. The structure of genistein and equol is very similar to that of estrogen, so both compete with estrogens to bind to the ER and modulate ER function (Figure 2) [14,84].

The affinity of genistein and equol for binding to ER-β is similar to the affinity of 17-β estradiol (E2) for this receptor, although the concentration of genistein required to induce transcription is 10^4^ times higher compared to E2 [84].

There also is a clear link between the ER-β and the androgen receptor (AR): activation of the ER-β downregulates the AR, which results in a reduced response of prostate tissue to androgen stimulation [17,85,86]. In turn, this effect causes reduced production of the prostate-specific antigen (PSA) [87]. In the case of androgen-sensitive prostate cancer cells, low concentrations (0.1–5 µM) of genistein are sufficient to lower the PSA level, whereas in androgen-independent cell lines, concentrations should be markedly higher (10–50 µM) [38,88]. This isoflavone-induced downregulation of the AR was also demonstrated in animal models (diet containing 100–500 mg/kgBW genistein daily), which in turn reduced tumor growth [17,89,90,91].

Equol has a unique ability to bind dihydrotestosterone (DHT), thus sequestering DHT and preventing binding to the AR, which has anti-androgenic effects [92]. Via a ubiquitin ligase (Skp2) and a proteasomal pathway, equol also causes AR degradation (=ubiquitination) [24]. In turn, genistein also causes AR degradation via ubiquitination, but with the help of heat shock proteins (HSP), including HSP90 [18,19].

In addition to all these described mechanisms of action via AR interaction at the cellular level, there may also be an effect on AR gene expression. In particular, genistein in vitro (10 µM) is thought to be effective in this way, with a decrease in AR mRNA [5]. However, in a study with male rats, equol at a dose of 100 to 250 mg/kgBW/day was found to be unable to change AR mRNA expression in the prostate [20].

## 4. Inhibition of Cancer Cell Growth

In human prostate cancer cells, genistein was able to inhibit cell growth independent of AR status, and this was seen in both the androgen-independent cell lines PC-3 and DU-145 and in the androgen-sensitive LNCaP line [40,45,47,48,91,93,94,95,96]. An important mechanism induced by high doses of genistein (>10 µM) is the inhibition of growth factor tyrosine kinase (TK) activity [25]. Inhibitors of these TKs nowadays play a prominent role in the treatment of several malignancies [97], and TKs might be interesting targets for PCa-targeted therapies [6]. Concerning PCa, receptor-mediated TK activation is considered to be one of the mechanisms for acquiring the androgen-independent (or castration-refractory) status [98,99]. However, in in vitro experiments, the dose of genistein needed to achieve this effect reached the upper limit of physiologically attainable doses (>10 µM) [25], a situation that is not realistic in vivo [10].

PCa cells often have an increased expression of the ErbB receptor family (proteins), such as the epidermal growth factor receptor (EGFR or ErbB-1), ErbB-2 (also called HER2), and ErbB-3 (also named HER3) [25]. Genistein at a higher dose (100–200 µM) is a potent inhibitor of the EGFR in the androgen-independent DU-145 cell line [26]. In animal models, the inhibition of the expression of both EGFR and ErbB-2 receptors by genistein (0.05–1 mg/g diet) was demonstrated [27].

Insulin growth factor 1 (IGF-1) is also thought to play an important role mainly via promoting progression and metastasis but also blocking apoptosis [100,101,102]. TKs also play a role in this, as they are activated when IGF-1 binds to its membrane receptor. As a result, the insulin receptor substrate (IRS-1) is phosphorylated [32]. In turn, PI3K/AKT and RAS/MAPK are activated, resulting in cell proliferation. This IGF-1-stimulated cell growth was inhibited by genistein in PC-3, LNCaP, and DU-145 cell lines at rather average doses (25–40 µM) [28,29,30]. Moreover, genistein inhibits the phosphorylation of other mediators such as glycogen synthase kinase-3β (GSK-3β), Src, FOXO3a, Akt, and p70S6k, leading to the downregulation of AR [21,22,23].

FOXO (forkhead box O) proteins can suppress tumors, but these may themselves be inhibited through mitogen-activated protein kinase (MAPK)-mediated phosphorylation [103,104]. This phosphorylation is inhibited by genistein, equol, and daidzein, which can increase FOXO proteins [29,31].

Another pathway involved in the progression of PCa is the Wnt/β-catenin pathway. Upon the presence of the Wnt ligand, cytoplasmic β-catenin is phosphorylated and freed from its complex. At the level of the cell nucleus, binding to the transcription factor T-cell factor-4 (TCF-4) occurs, resulting in the activation of transcription of genes responsible for cell proliferation (c-Myc and cyclin D1) [33]. Blocking this pathway in PC-3 cells with genistein (100 µM) resulted in the marked suppression of PCa cell growth [34,35].

Poly(ADP-ribose)polymerase (PARP)-inhibitors are a new kid on the block in the treatment of metastatic PCa by acting on apoptotic cell death. In PC-3 and LNCaP cells, genistein induces cleavage of PARP [48]. Cleavage occurs by caspase 3, on which genistein acts specifically, as demonstrated in PC-3 cell lines [36]. In these PC-3 cells, genistein, even at doses of 50 µM, decreased the activity of Akt kinase and reduced phosphorylation of the Akt protein when compared to PC-3 cells that were not treated with genistein [38]. This reduced Akt phosphorylation leads to the decreased antiapoptotic function of the protein. This gives rise to the hypothesis that genistein acts as an initiator of apoptotic cell death, at least in PC-3 cells. Additionally, PC-3 cells treated with genistein showed a reduction in mRNA levels of survivin and protease-activated receptor 2 (PAR-2) that delay apoptosis. In contrast, mRNA levels for elafin were increased, which increases apoptosis [37].

Two key proteins involved in maintaining balance in cellular life are Bcl-2 and Bax. Bcl-2 inhibits cellular apoptosis, while Bax stimulates it via stimulation of the mitochondria with the release of cytochrome C and activation of caspases. Genistein (25 µM) stimulates Bax and suppresses Bcl-2, giving a stronger ratio for Bax and inducing apoptosis [38].

Genistein can also induce apoptosis through interfering with the proteasome. This is a protein complex that degrades proteins that are no longer needed or damaged via proteolysis. Thus, proteins that promote cell cycle regulation can be degraded, leading to apoptosis [39]. A simultaneous accumulation of ubiquitinated proteins was seen, including the cyclin-dependent kinase (CDK) inhibitor p27, the inhibitor of nuclear factor-Kβ (NF-Kβ), and the Bax protein.

Nuclear factor-Kβ (NF-Kβ) are transcription factors that, when activated, can protect against apoptosis. They do this via binding to the so-called Kβ sites of DNA (5′-GGGRNYY YCC-3′), and this process is mediated by the IKB protein [42,43,105]. Genistein appears to inhibit the binding of NK-Kβ to DNA, which could be demonstrated in the different prostate cancer cell lines at a moderate dose of 50 µM [37,40,41]. This inhibition is based on the inhibition of the phosphorylation of IKB. Consequently, the effect of NK-Kβ on DNA is prevented, and protection from apoptosis is countered [41]. Moreover, there is a link between NF-KB and the Akt pathway. Akt enhances the degradation of IKB and thereby induces NF-Kβ activity [106]. As mentioned above, genistein has been demonstrated to inhibit the Akt signaling pathway and NF-Kβ activation through this mechanism [40].

Autophagy is viewed as a variant of programmed cell death in which cellular components are degraded by lysosomes [107]. In autophagy, the mammalian target of rapamycin (mTOR) has a signaling function, and it inhibits autophagy [108]. Soy isoflavones are able to suppress this mTOR signaling, and thus, autophagy is not inhibited, which was demonstrated in LNCaP and 22Rv1 PCa cells [44].

Furthermore, genistein (50 µM) also appears capable of downregulating telomerase reverse transcriptase, c-Myc RNA, and MDM2 oncogene, as was seen in the PCa cells DU-145 and LNCaP [45,46].

## 5. Effects on Cell Cycle Regulation

In PCa cells in culture, genistein inhibited growth with the arrest of the G2/M cell cycle, which was related to dose (5–50 µM). Simultaneously, there was downregulation of cyclin B1, upregulation of the growth-inhibitory protein p21WAF1, and induction of apoptosis [48]. In the androgen-independent cell line DU-145, there was also genistein-induced inhibition of the adaptor protein Shc, resulting in the inhibition of extracellular regulated kinase (ERK)1/2 activation. This inhibition was dose-dependent (100–200 µM) and without alteration in protein levels [26]. The inhibition of cell growth was also found in androgen-independent PC-3 cells, as well as in the androgen-sensitive LNCaP cells [48].

Cyclin-dependent kinases (CDKs) and cyclins are regulatory switches that allow the cell to move through the different phases of the cell cycle (from G1 to S-phase and from G2 to M-phase). Genistein interferes with these control switches and causes cell cycle arrest at various concentrations (20–200 µM) [48,49,109,110]. In the LNCaP cell line, genistein induced the G1 cell cycle arrest through the upregulation of CDK inhibitors [47]. In PCa cells, genistein induced the G2/M cell cycle arrest, combined with an increase in p21 and p27 and a decrease in cyclin B1 and CDK4 [30,48,49]. A similar mechanism at similar concentrations was seen for equol in PC-3 cells, along with an induction of apoptosis through the upregulation of Fas ligand (Fas) and expression of proapoptotic Bim [31]. The decreased expression of cyclin B1 was seen along with an increase in P53 proteins in both LNCaP and PC-3 cells with genistein and daidzein, even at low doses (5–10 µM) [111].

## 6. Angiogenesis

Through angiogenesis, tumor cells attempt to grow and expand, and they do so via growth factors, such as vascular endothelial growth factor (VEGF), basic fibroblast growth factor (bFGF), and platelet-derived growth factor (PDGF) [112]. In addition, IL-8 also strongly stimulates angiogenesis, increasing the metastatic potential of PCa [113]. The expression of IL-8 is related to tumor aggressiveness and the Gleason score [114,115].

In PC-3 cells, isoflavones decreased the mRNA level of IL-8, while genistein (5–50 µM) inhibited VEGF expression at the hands of hypoxia-inducible factor-1α (HIF-1α) [50,51]. Furthermore, the mRNA level of VEGF receptors 1 and 2 (VEGFR1 and VEGFR2) in human endothelial cells was inhibited by genistein already at a low dose (2.5 µM) [52]. Even genistein is able to inhibit endothelial cell proliferation in a direct manner [53,116].

In LNCaP, PC-3, and DU-145 PCa cells, both genistein (40 µM) and daidzein (110 µM) were shown to directly inhibit a number of genes. These were genes encoding molecules involved in angiogenesis such as fibroblast growth factor 1 (FGF1), platelet-derived endothelial cell growth factor (ECGF1), fibroblast growth factor receptor 3 (FGFR3), platelet/endothelial cell adhesion molecule (CD31 antigen or PECAM1), IGF1, IL-1β, IL-6, IL-8, and CXC ligand 10 [30,50,53].

Transforming growth factor-β (TGF-β) acts as a tumor promoter in metastatic PCa, also through stimulating angiogenesis [117]. This occurs via the increased phosphorylation of matrix metalloproteinase type 2 (MMP-2) and mitogen-activated protein kinase p38 (MAPK p38). Genistein was able to completely suppress this process in PC-3 cells even at a very low concentration of 10 nM, corresponding to concentrations reached in the blood after dietary consumption [54].

Inhibitory effects on angiogenesis could also be observed for equol via similar pathways in bovine brain capillary endothelial cells (BBCE) [59]. This mainly involves the MAPK pathway through direct action on VEGF, FGF2, and extracellular regulated kinase (ERK) 1/2. A similar mechanism was also observed with equol in vitro in mouse epidermal cells, leading to tumors with smaller volumes [7,60].

## 7. Tumor Cell Invasion and Cancer Metastasis

Several phases are distinguished in metastasis, including epithelial-to-mesenchymal transition (EMT), destruction of the extracellular matrix (ECM), and mesenchymal-to-epithelial transition (MET) [118].

In IA8-ARCaP human PCa cells, genistein (dose-dependent 15–75 µM) was found to be able to inhibit invasive growth through reversing EMT [62]. AKR1C3 is an enzyme that specifically increases androgens in castration-resistant prostate cancer (CRPC) and metastatic PCa. Genistein inhibits metastasis through inhibiting AKR1C3, which acts as an EMT driver through the activation of ERK signaling. This was demonstrated not only in CRPC cell lines with different concentrations of genistein (0–100 μM) but also in a xenograft tumor mouse model fed with 100 mg/kgBW/day of genistein [63,64].

As previously mentioned, genistein, as well as daidzein and equol, inhibits the function of matrix metalloproteinases (MMPs). These are important enzymes in metastasis because they can degrade both the extracellular matrix and the basal membrane of cells. Genistein, daidzein, and equol can exert (in vitro) an inhibitory effect on MMP-2, MMP-9, and urokinase-type plasminogen activator and thus inhibit tumor invasion in DU145 cells, with a slightly higher concentration for equol (5–50 µM) versus daidzein and genistein (0.5–5 µM) [55,56]. The suppression of MMPs by genistein has been observed both in PC-3 and LNCaP cell lines in vitro, but also in vivo with orthotopically implanted human PC-3 cells in mice that reached blood concentrations similar to those measured in genistein-consuming men [57,119,120]. This occurs indirectly via inhibition of focal adhesion kinase (FAK), MAP kinase-activated protein kinase 2 (MAPKAPK2), and heat shock protein 27 (HSP27), two regulators of MAPK p38, by genistein [54,58,121,122,123].

Prostaglandins (PGs) can promote PCa cell development, and the enzymes cyclooxygenase-1 (COX-1) and COX-2 are involved in the production of these PGs [124,125]. COX-2 overexpression is observed in, among others, PCa, and this is associated with increasing tumor angiogenesis and invasion [126,127,128]. Soy isoflavones, including genistein at a dose of 10 µM, are known to inhibit this production of PGs [129]. Genistein also induces mRNA levels of 15-hydroxy prostaglandin dehydrogenase, which causes PG degradation [61].

Osteopontin (OPN) is a protein that plays a role in bone remodeling, and it may be more linked to the context of tumor growth and metastasis of PCa [130,131]. Genistein has an inhibitory effect on OPN mRNA levels, which was demonstrated both in vitro in PC-3 cells (genistein 50 µM) [23] and in vivo in Transgenic Adenocarcinoma Mouse Prostate (TRAMP) (genistein diet 250 mg/kgBW/day) [65].

In vivo data are indeed very promising, such as genistein reducing the migration of both PC-3 cells [132] and cell lines of PCa in rats, namely MAT-LyLu and AT-2 [133]. In SCID mice implanted with LNCaP cells, soy isoflavones were able to inhibit the disease from spreading to glands and lungs [134]. Genistein was found to affect metastasis in a mouse model after implantation of PC-3 cells in the prostate [57]. Treatment with genistein reduced the number of lung metastases but did not alter tumor growth. Other experiments in rats confirmed an overall increase in survival after a boosted diet with genistein (250 mg/kgBW/day) [135].

## 8. Antioxidant Effect

Free radicals and ROS (Reactive Oxygen Species) are continuously generated during normal oxygenation. However, in stress situations, due to exposure to noxious agents or pathologic processes, there is increased production of both free radicals and ROS, which are known for their high toxicity on both cells and enzymes.

Soy isoflavones, and especially equol, have strong antioxidant properties. They do not act as antioxidants themselves, but through acting on signaling pathways, they cause a modification in the expression of cellular enzymes (such as superoxide dismutase (SOD), catalase, and glutathione peroxidase) and protect the cells from free radicals and ROS [66,67,68,69].

In DU 145 cells, genistein at a dose of 10 µM increased antioxidant enzymes via AMP-activated protein kinase (AMPK) and phosphatase and tensin homolog deleted from chromosome 10 (PTEN) pathways [70]. There was also an increase in manganese SOD and catalase, which further suppressed the level of ROS. Moreover, genistein was able to subdue the production of nitric oxide (NO), a free radical, through suppressing NO synthase (NOS).

Presumably, equol has a greater antioxidant activity than genistein and daidzein due to a more extensive inhibition of NO production. It is assumed that equol inhibits the production of NO and the expression of the inducible nitric oxide synthase (iNOS) gene through blocking Akt and NF-kappaB [71]. This could be demonstrated both in vitro in RAW 264.7 cells and in vivo through the administration of equol (20–50 mg/kg intraperitoneal) in isolated peritoneal adherent cells from mice treated with lipopolysaccharide, which causes increased nitrite levels.

## 9. Anti-Inflammatory Effect

The functioning of the immune system in cancer is complex and dual. Inflammation is basically a reaction of immune cells in the host against cancer cells. On the other hand, those cells release cytokines that can, however, promote angiogenesis and, therefore, progression. In turn, tumor-associated macrophages (TAMs) can release various substances, such as inflammatory mediators, growth factors, cytokines, and proteolytic enzymes that are important in metastasis. It is precisely these TAMs on which genistein exerts its most important cancer inhibitory effect. Genistein at a dose of 100 µM decreased the number of TAMs, which reduced the density of blood vessels and, thus, the tumor in the R3327 MAT-Lu cell line. Indirectly, this occurred via a decrease in TNF-α and granulocyte-monocyte colony-stimulating factor by genistein [136]. This same decrease in TNF-α and expression of TNF-α mRNA was also observed for equol in mouse macrophages via an intermediate pathway of NF-kappaB blockade [72].

Interference with interleukin-10 (IL-10) has also been established for genistein (100 µM) in PCa cells, suggesting that it also has an inhibitory effect on inflammation [73]. Interference with inflammatory mechanisms was demonstrated for equol (10 µM) and genistein (20 µM), but not for daidzein, via the inhibition of prostaglandin E2 in activated macrophages in the RAW 264.7 cell model [74].

## 10. Epigenetics

Genistein inhibits DNA methylation and histone modifications, two key factors of gene regulation [75,76,77,78]. For both genistein (40 µM) and daidzein (110 µM), a reduction in the methylation of, among others, the tumor suppressor BRCA1 gene in PC-3 and DU-145 cells was demonstrated [75]. Specific to genistein, such reduced methylation was also seen in other tumor suppressor genes, such as B-cell translocation gene 3 (BTG3) and Ras association domain family 1 (RASSF1A) [76]. Methyl-binding domain proteins and DNA methyltransferase enzymes were also inhibited in terms of expression and activity by genistein [77,78].

Genistein regulates microRNAs (miRNAs), small endogenous RNAs that regulate gene expression [79,80,81,137,138]. Genistein (40 µM) and daidzein (110 µM) decrease the expression of miRNAs, which are involved in cell growth and survival in PC-3, DU145, and LNCaP PCa cells [79]. This occurs via a reversal of methylation of the promoter of the epigenetically repressed miRNA. A decrease in the methylation of the promoter sequence of miR-29a and miR-1256, in turn, causes inhibition of two oncogenes, tripartite motif-containing protein 68 (TRIM68) and phosphoglycerate kinase 1 (PGK-1), resulting in inhibitory growth and decreased invasion in PCa cells [80]. This was also demonstrated in vivo: multiple miRNAs were differentially expressed in the plasma of mice orally administered daidzein 20 mg/kgBW/day [81].

Equol is able to inhibit cancer cell proliferation in in vitro studies at a dose of 50–100 µM through inducing the polyadenylation of small nucleolar RNAs (snoRNAs) via a poly(A) polymerase associated domain containing 5 (PAPD5)-dependent pathway [82].

## 11. Conclusions

Both in vivo and in vitro preclinical data show interesting biological inhibitory influences and interactions with numerous carcinogenic and metastatic pathways. This is promising and could assign soy isoflavones a chemopreventive role in prostate cancer. However, this evidence is not yet confirmed through clinical trials, and more research is needed. Different aspects of bioavailability-influencing factors need to be taken into account in well-outlined clinical trial protocols.

## Figures and Tables

**Table 1:** Anti-prostate cancer molecular mechanisms of isoflavones genistein (G), daidzein (D), and equol (E).

Effect	Mechanism	G	D	E	References
Receptor-mediated carcinogenesis	Downregulation AR	x			[17,18,19,20]
			x		[7,21,22,23]
				x	[24]
Inhibition of cancer cell growth	↓TK	x			[25]
	↓EGFR or ErbB-1	x			[26]
	↓ErbB-2 or HER2	x			[27]
	↓IGF1 (PI3K/AKT and RAS/MAPK pathway)	x			[28,29,30]
	↓phosphorylation Src, AKT, GSK-3β, FOXO3a and p70S6k	x			[21,22,23]
	↓phosphorylation FOXO, AKT		x	x	[29,31]
	↓IRS-1	x			[32]
	↓Wnt/β-catenin	x			[33,34,35]
	↑caspase3	x			[36]
	↓survivin, ↓PAR-2, ↑elafin	x			[37]
	↑Bax, ↓Bcl-2	x			[38]
	↓proteasome	x			[39]
	↓NF-Kβ	x			[37,40,41]
	↓phosphorylation IKB	x			[42,43]
	↓mTOR	x			[44]
	↓TRT, ↓c-Myc, ↓MDM2	x			[45,46]
Cell cycle regulation	G1 arrest	x			[30,47,48,49]
	G2/M arrest	x	x	x	[31]
	↓cyclin B1	x			[48]
			x	x	[31]
	↑p21WAF1	x			[48]
	↓adaptor protein Shc, ↓ERK1/2	x			[26]
	↓CDK1			x	[31]
	↓CDK4	x			[48]
	↑p21, ↑p27	x			[39,48]
				x	[31]
	↑FasL, ↑Bim			x	[31]
Angiogenesis	↓VEGF,↓ HIF-1α	x	x		[50,51]
	↓VEGFR1, ↓VEGFR2	x			[52]
	↓ECGF1, ↓FGF1, ↓IGF1, ↓ FGFR3, ↓CXC, ↓ IL-1β, ↓IL-6, ↓IL-8, ↓ Ligand 10, ↓PECAM1	x	x		[30,50,53]
	↓TGF-β, ↓MMP-2, ↓ MAPK p38	x			[54]
Anti-metastatic	↓urokinase-type plasminogen activator, ↓MMP-2, ↓MMP-9	x	x	x	[55,56]
	↓MAPKAPK2, ↓ HSP27, ↓FAK	x			[54,57,58]
	↓VEGF, ↓FGF2, ↓MEK (or MAPK)1/2, ↓ERK1/2			x	[7,59,60]
	↓PG, ↓ COX-1, ↓ COX-2	x			[61]
	↓EMT	x			[62]
	↓AKR1C3	x			[63,64]
	↓OPN	x			[23,65]
Antioxidant	↑SOD, ↑catalase, ↑glutathione peroxidase	x	x	x	[66,67,68,69]
	↑AMPK, ↑PTEN, ↓NO, ↓NOS	x			[70]
	↓NO, ↓AKT, ↓NF-κB, ↓ TNF-α, ↓iNOS			x	[71,72]
Anti-inflammatory	↓TAM, ↓ TNF-α, ↓GM-CSF	x	x	x	[69]
	↓IL-10	x			[73]
	↓PG-E2	x		x	[74]
Epigenetics	↓methylation (e.g., BRCA1)	x	x		[75]
	↓methylation (BTG3, RASSF1A)	x			[76]
	↓methyl binding domain proteins	x			[77]
	↓DNA methyl transferase enzymes	x			[78]
	↓miRNA	x	x		[79]
	↓miR-29a, miR-1256, TRIM68, PGK-1	x			[80]
	miRNA		x		[81]
	snoRNAs			x	[82]

## Data Availability

No new data were created or analyzed in this study. Data sharing is not applicable to this article.
